# Emergence of Dalbavancin, Vancomycin, and Daptomycin Cross-resistance in MRSA During Long-term LVAD Suppression With Vancomycin Followed by Dalbavancin: Genomic Insights and Synergy With Cefadroxil

**DOI:** 10.1093/ofid/ofag074

**Published:** 2026-02-16

**Authors:** Erica J Stohs, Bryan T Alexander, Paul D Fey, Melanie C L Foreman, Libin Xu, Kelsi Penewit, Adam Waalkes, Stephen J Salipante, Brian J Werth

**Affiliations:** Division of Infectious Diseases, Department of Medicine, Creighton University Medical Center, Omaha, Nebraska, USA; Department of Pharmaceutical & Nutrition Care, Nebraska Medicine, Omaha, Nebraska, USA; Department of Pathology, Microbiology, and Immunology, University of Nebraska Medical Center, Omaha, Nebraska, USA; Department of Pharmacy, School of Pharmacy, University of Washington, Seattle, Washington, USA; Department of Medicinal Chemistry, School of Pharmacy, University of Washington, Seattle, Washington, USA; Department of Laboratory Medicine and Pathology, School of Medicine, University of Washington, Seattle, Washington, USA; Department of Laboratory Medicine and Pathology, School of Medicine, University of Washington, Seattle, Washington, USA; Department of Laboratory Medicine and Pathology, School of Medicine, University of Washington, Seattle, Washington, USA; Department of Pharmacy, School of Pharmacy, University of Washington, Seattle, Washington, USA

**Keywords:** chronic infection, dalbavancin resistance, daptomycin nonsusceptible, driveline infection, lipoglycopeptide cross-resistance

## Abstract

We present a case in which vancomycin and dalbavancin exposure preceded emergence of nonsusceptibility to dalbavancin, vancomycin, and daptomycin in a strain of methicillin-resistant *Staphylococcus aureus* in the setting of a left ventricular assist device (LVAD) infection. We characterized 6 related but genetically unique isolates collected over more than 1 year of recurrent therapy and found that the most resistant isolate acquired multiple *walK*-related mutations, which has been previously implicated in dalbavancin resistance with vancomycin and daptomycin cross-resistance. Using time-kills at subinhibitory exposures, we found that cefadroxil is synergistic with dalbavancin against the susceptible and resistant strains. This is the first report of dalbavancin nonsusceptibly associated with an LVAD infection, but dalbavancin resistance has been documented previously in association with treatment of endovascular infections. Combination therapy with synergistic and orally bioavailable agents like cefadroxil may be a reasonable strategy to enhance activity and possibly diminish emergence of resistance to dalbavancin.

Left ventricular assist device (LVAD) infections secondary to *S. aureus* are challenging to treat, especially when exchange of the infected device is not feasible. Antimicrobial therapy in this setting is typically suppressive rather than curative in nature, and durations of therapy can be extensive, which increases the risk of drug interactions, adverse effects, and resistance emergence. The long-acting lipoglycopeptide dalbavancin is an appealing option due to its potent activity against methicillin-resistant *Staphylococcus aureus* (MRSA), prolonged dosing interval, and excellent safety profile. Reports of clinical experience with this approach are limited, but favorable outcomes have been reported in at least 18 cases [1–4]. Breakthrough infections have also been reported, but dalbavancin resistance selection has not been described in patients with LVAD infections from MRSA [4]. Emergence of dalbavancin resistance on therapy remains rare and has primarily involved complicated endovascular sources [5–8]. Synergistic combinations of antimicrobials can enhance bacterial killing and reduce the likelihood of resistance emergence and are a standard approach for challenging, biofilm-related device infections. Dalbavancin has been shown to be synergistic with several beta-lactams [9–11], but there is limited evidence supporting other combinations including orally bioavailable antibiotics. Here we report the first case in which dalbavancin resistance with cross-resistance to vancomycin, daptomycin, and oritavancin was selected by dalbavancin exposure in a patient with LVAD infection. Furthermore, we characterized the resistant isolates by whole-genome sequencing and evaluated orally bioavailable companion antibiotics for synergy with dalbavancin using time-kills.

## CASE REPORT

### Clinical Course

A male in his mid-40s with ischemic cardiomyopathy requiring an implantable cardioverter defibrillator (ICD) and left ventricular assist device (LVAD; HeartMate II) developed a MRSA driveline exit-site infection 26 months after LVAD placement. After a 6-week course of vancomycin, he was transitioned to oral antimicrobial suppressive therapy (AST) with doxycycline. Over the following 13 months, he developed 4 episodes of recurrent MRSA bacteremia despite modifying AST from doxycycline monotherapy to combination doxycycline plus rifampin, and finally to trimethoprim/sulfamethoxazole. Each episode of bacteremia was treated with vancomycin for a minimum of 6 weeks with therapeutic drug monitoring by our institution's outpatient parenteral antimicrobial therapy (OPAT) team. By the third episode of bacteremia at 3 years post-LVAD, new ICD lead vegetations were noted on transesophageal echocardiogram (TEE), and daptomycin was initiated for therapy for this episode. Unfortunately, he developed daptomycin-induced rhabdomyolysis (creatine kinase peak, 32 000 U/L) 2 weeks into therapy, precluding its future use.

**Figure 1.**
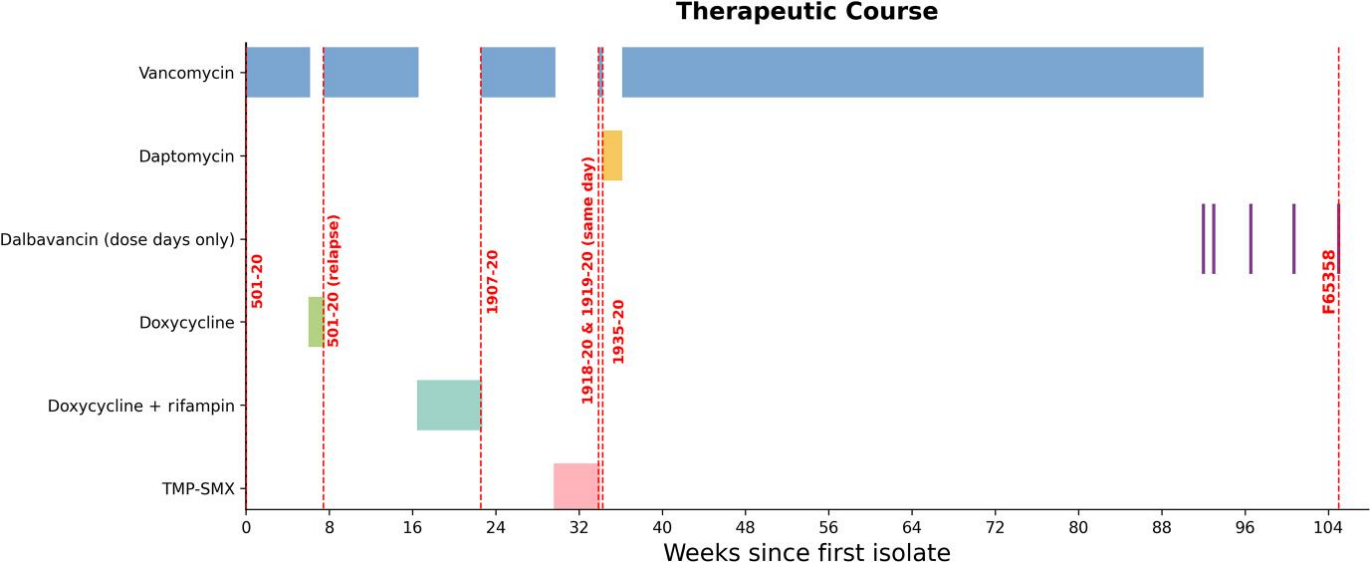
The timeline illustrates the therapeutic course for this patient from the initial episode of bacteremia with MRSA strain 501–20 until the emergence of the dalbavancin-, daptomycin-, and vancomycin-nonsusceptible MRSA strain (F65358). Horizontal bars represent systemic antimicrobial regimens, and vertical dashed red lines mark the timing of cultured isolates. Abbreviations: MRSA, methicillin-resistant *Staphylococcus aureus*; TMP-SMX, trimethoprim-sulfamethoxazole.

Due to limited AST options, the patient was maintained on intravenous vancomycin monitored by the OPAT team for 1 year, at which time he was deemed not a transplant candidate at 4.5 years post-LVAD, for non-infectious-related reasons. We then offered a plan for dalbavancin AST as an alternative, with the patient's agreement. He was initially administered dalbavancin 1500 mg intravenously (IV) weekly for 2 doses, then 4 weeks following the second dose he began AST with 1000 mg IV every 4 weeks. Three months into dalbavancin suppression, the patient developed low-grade fever and low-flows with the LVAD, indicating possible sepsis. Computed tomography of the chest indicated extension of a fluid collection from the driveline to the inferior outflow cannula. Blood cultures were obtained in the infusion center immediately before his regularly scheduled dalbavancin infusion, which revealed vancomycin-intermediate *Staphylococcus aureus* (VISA; isolate F65358) (Table 1). The patient was briefly admitted but declined any alteration of his antibiotic regimen and left against medical advice. This clinical course is illustrated in Figure 1.

**Table 1.** MIC Values in µg/mL; PAP AUC Ratio With Mu3 ≥0.9 Was Considered Positive for hVISA

	Cefadroxil	Dalbavancin	Daptomycin	Doxycycline	Linezolid	Nafcillin	Oritavancin	Rifampin	Trimethoprim	Vancomycin	Vancomycin PAP AUC Ratio
501–20^a^	≥64	<0.0039	0.125	2	1	8	0.125	<0.01	1	1	0.52
1907–20	R	<0.0039	0.0625	S	1	4	0.125	>16	S	1	0.54
1918–20	R	0.0156	0.125	S	1	8	0.5	>16	S	2	0.79
1919–20	R	0.0156	0.125	S	1	16	0.5	>16	S	2	0.92-hVISA
1935–20	R	0.0156	0.125	S	1	32	0.5	>16	S	1	0.41
F65358	32	1	2–8	0.25	1	2	2	>16	2	8	VISA

Abbreviations: AUC, area under the curve; hVISA, heterogeneous vancomycin-intermediate *Staphylococcus aureus*; MIC, minimum inhibitory concentration; PAP, population analysis profile; VISA, vancomycin-intermediate *Staphylococcus aureus*.

^a^Two isolates recovered between isolates 501–20 and 1907–20 were genetically identical to 501–20 and have been removed from the table.

In subsequent goal-of-care discussions, the patient opted for alternative outpatient therapies, initially with oral linezolid for 3 months (well tolerated), resumption of dalbavancin 1000 mg every 3 weeks, plus oral trimethoprim/sulfamethoxazole for ∼3 months, and finally oritavancin 800 mg IV weekly was substituted for dalbavancin in the combination due to a dalbavancin shortage (∼4 months). While he remained bacteremia-free and clinically stable during that period, he ultimately developed gastrointestinal bleeding due to erosion of the LVAD into the stomach and died of complications 2 months later.

### Patient Consent

This study does not include factors necessitating patient consent. All patient information has been anonymized in accordance with institutional policy and applicable privacy standards. Institutional review board approval was not required because the report does not meet the definition of human subjects research.

## METHODS

### Susceptibility Testing

Susceptibility testing was performed by MicroScan WalkAway (Beckman Coulter, Brea, CA, USA) and/or Etest at the Nebraska Medical Center as part of standard patient care. Additionally, minimum inhibitory concentrations (MICs) to dalbavancin, daptomycin, vancomycin, oritavancin, nafcillin, cefadroxil, trimethoprim, doxycycline, and rifampin were confirmed in duplicate by manual broth microdilution per Clinical Laboratory Standards Institute guidelines for the initial parent isolate and the dalbavancin-nonsusceptible isolate [12]. Vancomycin susceptibility by the modified population analysis profile method was performed on all isolates with vancomycin MIC ≤2 mg/L as previously described to test for heterogeneous VISA (hVISA) [13].

### Whole-Genome Sequencing

Whole-genome sequencing (WGS) library preparation, sequencing, and analysis were performed on 8 of the patient's blood isolates as previously described [14], with mapping conducted against Genbank accession CP049435.1 as the most closely matched reference genome. De novo genome assemblies were examined for *blaZ* and PVL carriage using BLASTx, established SCCmec type using SCCmecFinder 1.2 (https://cge.food.dtu.dk/services/SCCmecFinder/), and *agr* allele using AgrVATE (https://github.com/VishnuRaghuram94/AgrVATE). Sequence data from this project are available from the NCBI Sequence Read Archive (Accession PRJNA1345763).

### Phenotypic Assessment

We evaluated the isolates for *agr* function by the delta-hemolysis assay as described previously [15]. To assess growth rate, we inoculated 200 µL of tryptic soy broth with 6-log_10_ CFU/mL of each isolate in sextuplicate and measured the OD_600_ every 10 minutes for 30 hours while incubating at 37°C with shaking on a BioTek Synergy H1 microplate reader (Agilent). Colonies were inspected visually to compare size and pigmentation.

### Time-kill Synergy Studies

To evaluate whether dalbavancin was synergistic with orally bioavailable antimicrobials, we performed time-kills as previously described [9]. We prioritized orally bioavailable agents with standard dosing frequencies ≤ twice-daily to capitalize on the key advantage of dalbavancin—its reduced dosing frequency. We combined half-MICs of dalbavancin with cefadroxil, trimethoprim, or doxycycline in Mueller-Hinton broth supplemented with 0.002% polysorbate-80. Cefadroxil was selected over the more commonly prescribed cephalexin because of its longer half-life and reduced standard dosing frequency. Synergy was defined as a ≥2-log_10_ CFU/mL increase in killing compared with the most active single agent. Antagonism was defined as a ≥1-log_10_ CFU/mL increase in survival compared with the most active single agent. All other interactions were considered indifferent. As cefadroxil MICs were greater than the peak plasma concentration of a 1000-mg dose of cefadroxil (35 mg/L), the cefadroxil concentrations were capped at 35 mg/L to prevent overestimation of activity [16].

## RESULTS

### Susceptibility Testing, Whole-Genome Sequencing, and Phenotypic Assessment

**Figure 2.**
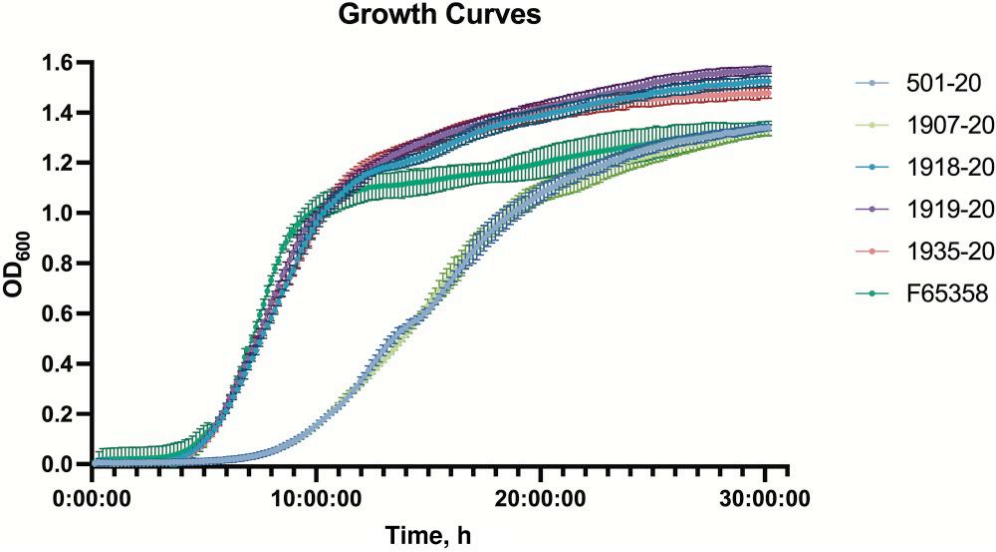
Growth curves of our 6 clinical isolates in tryptic soy broth at 37°C with shaking, reported as OD_600_ over 30 hours. Data points represent the mean OD_600_ of 6 replicate cultures for strains 501–20, 1907–20, 1918–20, 1919–20, 1935–20, and F65358, with error bars indicating standard deviation.

The strains isolated from the patient's blood are summarized in Tables 1 and 2. The initial isolate (501–20) was MRSA susceptible to vancomycin, daptomycin, dalbavancin, oritavancin, rifampin, doxycycline, linezolid, and trimethoprim/sulfamethoxazole. All isolates were resistant to beta-lactams other than ceftaroline, fluoroquinolones, macrolides, and clindamycin and were susceptible to linezolid, doxycycline, and trimethoprim/sulfamethoxazole. Isolates 501–20 and 1907–20 carried preexisting polymorphisms in *rpsJ, rsgA*, and FPP18_09955, which either reverted to wild-type or were replaced by alternative variants in subsequent strains. All but 501–20 acquired resistance to rifampin via 1 or more mutations in *rpoB*. The final isolate, F65358, was nonsusceptible to vancomycin, daptomycin, dalbavancin, and oritavancin but became more susceptible to beta-lactams as predicted by the seesaw effect [9, 17]. Isolates collected between 501–20 and F65358 were increasingly resistant to vancomycin, as indicated by increasing population analysis profile (PAP)–area under the curve (AUC) ratios, with 1 strain, 1919–20, meeting criteria (PAP_AUC ratio with Mu3 ≥0.9) for hVISA. The exception to this pattern was the penultimate strain, 1935–20, which became vancomycin susceptible before the emergence of F65358 16 months later. Relative to the parent strain 501–20, F65358 accumulated 15 mutations including those in several genes previously associated with the emergence of lipo-, glyco-, and lipoglycopeptide nonsusceptibility: *walK*, *stp1*, *mprF*, *rpoB*, and *tcaA* [18–21]. All strains were negative for the *blaZ* gene, carried the *agr* group II allele, were SCCmec type II, and were PVL positive. Phenotypically all strains were delta-hemolysin negative, indicating *agr* dysfunction, and minimally pigmented, appearing grayish white. Initial isolates carrying mutations in *rsgA* and FPP18_09955 exhibited impaired growth rates and small colonies compared with more resistant isolates carrying other mutations but wild-type *rsgA* and FPP18_09955 (Figure 2).

**Table 2.** Gene Names Are Listed in the First Column and Strain Names are Listed Across the First Row in Chronological Order From Left (501–20) to Right (F65358)

Gene	Description	501–20^a^	1907–20	1918–20	1919–20	1935–20	F65358
*rpsJ*	30S ribosomal protein S10	K57E	K57E	K57E	K57E	D60Y	D60Y
*rsgA*	Small ribosomal subunit biogenesis GTPase	I151fs	I151fs	–	–	–	–
FPP18_09955	Aromatic acid exporter family protein	T107I	T107I	–	–	–	–
*rpoB*	RNA polymerase subunit beta	–	D471G; I527L	H481R	H481R	Q468L	D471Y; L488S
*cobB*	NAD-dependent protein deacetylase	–	S10P	–	–	–	–
*walH*	Cell wall metabolism regulator (WalKR system)	–	–	D195fs	D195fs	–	–
*arlS*	2-component sensor histidine kinase	–	–	N70fs	N70fs	–	–
*lukG*	Leukotoxin G component	–	–	T145A	–	–	–
*FPP18_09710*	XRE family transcriptional regulator XdrA	–	–	–	G132D	–	–
*deoB*	Phosphopentomutase (nucleotide metabolism)	–	–	–	–	L10fs	–
*clpC*	ATP-dependent Clp protease ATP-binding subunit	–	–	–	–	G288V	–
FPP18_03760	EMMY motif lipoprotein	–	–	–	–	Y92fs	Y92fs
*srrA*	Response regulator SrrA (anaerobic metabolism)	–	–	–	–	(upstream)	–
FPP18_11290	GNAT family N-acetyltransferase	–	–	–	–	(upstream)	–
FPP18_11545	YjiH family protein	–	–	–	–	A371P	–
*walK*	Sensor histidine kinase (WalKR system)	–	–	–	–	–	M213T; M559I
*mprf*	Bifunctional lysylphosphatidylglycerol flippase/synthetase	–	–	–	–	–	T345A
*capA*	Capsule biosynthesis protein A	–	–	–	–	–	E131K
FPP18_01895	NDxxF motif lipoprotein	–	–	–	–	–	I36V
*sle1*	N-acetylmuramyl-L-alanine amidase (cell wall hydrolase)	–	–	–	–	–	V5fs
*cxaR*	Unknown function	–	–	–	–	–	K7fs
*stp1*	Serine/threonine phosphatase	–	–	–	–	–	G119fs
*ypsA*	DUF1273 domain-containing protein	–	–	–	–	–	Q52fs
FPP18_11955	GNAT family N-acetyltransferase	–	–	–	–	–	L64S
*TcaA*	Teicoplanin resistance-associated protein	–	–	–	–	–	I59fs

Variants identified by amino acid change, frameshift (fs), or whether the mutation occurred upstream of the translated portion of the gene.

^a^Two isolates recovered between isolates 501–20 and 1907–20 were genetically identical to 501–20 and have been removed from the table.

## TIME-KILLS

**Figure 3.**
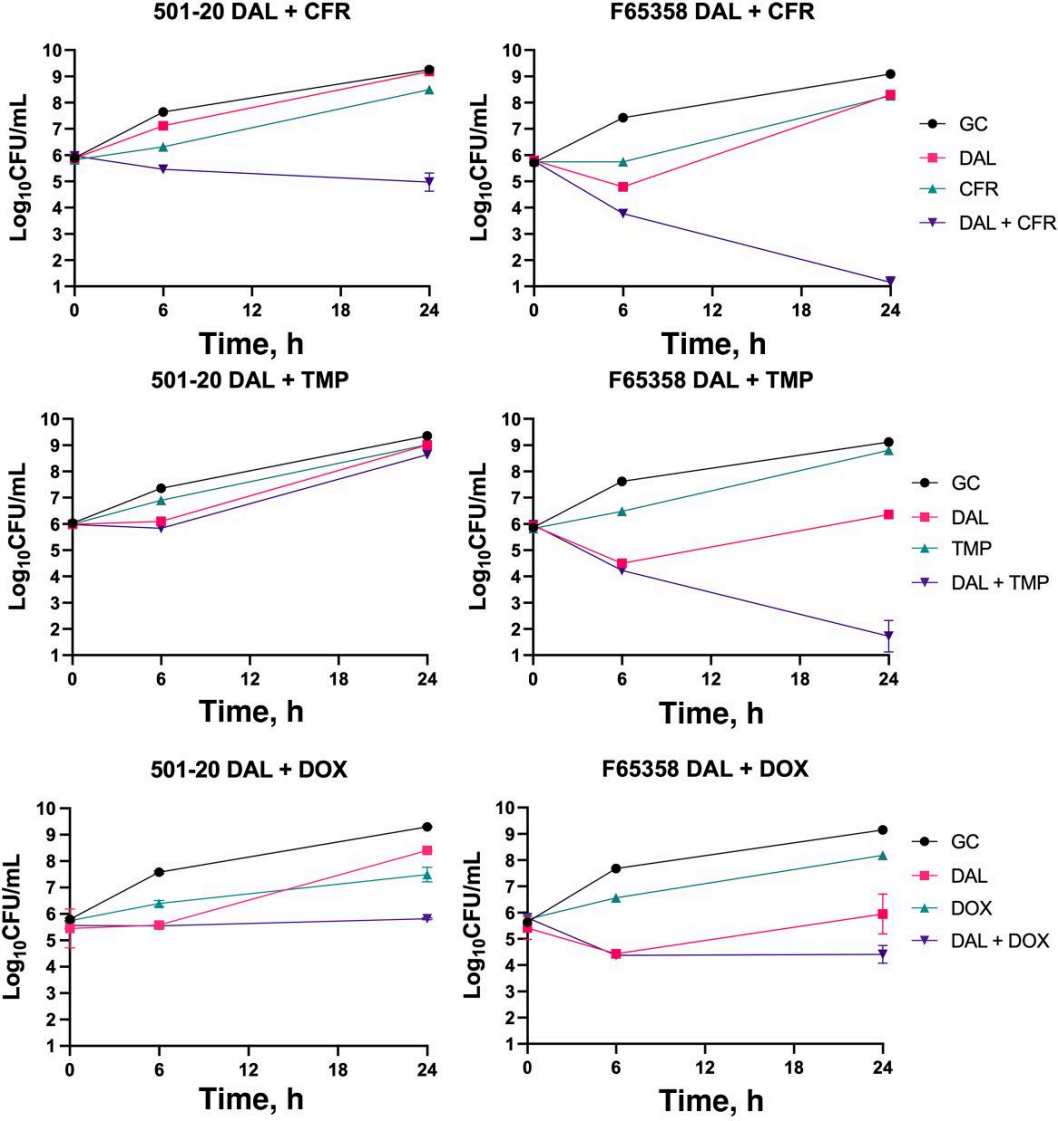
Time-kill curves demonstrating synergy between DAL and CFR for both susceptible (501–20) and resistant (F65358) strains. TMP was synergistic with DAL against F65358 but not 501–20. DOX was nonsynergistic with DAL against either strain. Abbreviations: CFR, cefadroxil; DAL, dalbavancin; DOX, doxycycline; TMP, trimethoprim.

The time-kill data are illustrated in Figure 3. The combination of dalbavancin plus cefadroxil was synergistic against both the parent strain (501–20) and the final dalbavancin-nonsusceptible strain (F65358) at subinhibitory concentrations and led to an average increase in bacterial killing of 5.31 log_10_ CFU/mL compared with the most active single agent. Dalbavancin plus trimethoprim was synergistic against F65358 but not 501–20, and dalbavancin plus doxycycline improved activity by <2 log_10_ CFU/mL, so it was considered indifferent against both. No antagonism was observed with any of the combinations tested.

## DISCUSSION

This case illustrates the challenges associated with treating LVAD infection. Because LVAD exchange is often not possible, clinical cure is often an unrealistic goal. Suppressive therapy in the absence of source control can prolong life and reduce morbidity, but the goals of therapy and optimal treatment strategies are often unclear, and resistance is often inevitable. Dalbavancin is an attractive option for suppression because of its potent activity against gram-positive pathogens and its ease of administration.

The DOTS trial found that dalbavancin was noninferior to 4–8 weeks of standard therapy for complicated *S. aureus* bacteremia, but the investigators excluded patients with retained hardware where source control was not feasible [22]. Observational data suggest that dalbavancin dosed every 2–8 weeks improved clinical stability and quality of life in patients with prosthetic joint, vascular graft, and endovascular infections who were candidates for indefinite suppressive therapy [23, 24]. Limited data were available to inform dosing recommendations for dalbavancin for AST at the time of this case, and either more frequent dosing or combinations might be warranted with vascular sources of infection, especially where prosthetic material is involved. Emerging data now point to therapeutic drug monitoring as a promising approach to guide dalbavancin AST dosing [25, 26]. Dalbavancin nonsusceptibility has been associated with endovascular infections with poor source control and prolonged therapy, including in the 1 instance of resistance emergence on therapy in the DOTS trial, where a patient with vertebral osteomyelitis evolved a vancomycin- and daptomycin-nonsusceptible isolate [6, 7, 22]. While dalbavancin resistance is still rare, it does tend to cross-select for vancomycin and daptomycin nonsusceptibility, which complicates sequential therapy options [5–7, 19, 27].

A commonly observed phenomenon among glycopeptide-nonsusceptible isolates is that many of them acquire fitness deficits as they become less susceptible [28]. Paradoxically, 501–20 and 1907–20 exhibited the slowest growth and smallest colonies. We suspect that this finding is related to their carriage of an *rsgA* frameshift variant, which reverted to wild-type among the later-emerging strains with larger colony sizes and faster growth. RsgA, sometimes called YjeQ, is a GTPase important for ribosomal biogenesis, and *rsgA* knockouts are known to have impaired growth [29]. We hypothesize that slow-growing, ribosome-limited populations could be less susceptible to many antibiotics due to slower metabolic rate [30] but that the subsequent emergence of more efficient drug-resistance variants could allow for reversion of growth-limiting mutations, improving bacterial fitness.

A common approach to managing biofilm-associated device infections with poor source control is to add an additional antimicrobial to enhance activity and reduce the likelihood of resistance. In this case, the patient received primarily sequential therapy where vancomycin was given at the onset of a bacteremia episode and eventually deescalated to a single oral agent, although oral doxycycline + rifampin was used for several weeks. Existing literature on synergistic combinations with dalbavancin have focused on agents that are intravenously administered multiple times daily, which diminishes the value of using dalbavancin in the first place. We performed our time-kills to assess for synergy with orally bioavailable drugs with standard dosing frequencies of once or twice daily to offer rigorous but practical evidence. Our time-kill data suggest that trimethoprim + dalbavancin was indifferent against the initial isolate (501–20) but synergistic against the least susceptible isolate (F65358), which is consistent with the suppression of bacteremia for >3 months while the patient was on trimethoprim/sulfamethoxazole and dalbavancin. Our time-kills show that doxycycline was not synergistic with dalbavancin against either strain, but it was also not antagonistic, and all isolates remained susceptible to doxycycline despite its use multiple times. As we only performed standard time-kills with planktonic cells, it is possible that we have underestimated how effective doxycycline would be against biofilm-embedded cells as doxycycline is often used as AST for biofilm-associated infections. Nevertheless, its low plasma concentrations and lack of clear synergy make it difficult to recommend this strategy for a chronic LVAD infection. Of the drugs tested, cefadroxil offered the most reliable and robust synergy with dalbavancin against both strains. While these data are promising, additional work is required to determine the optimal timing, duration, and dosing of this combination when it is considered for challenging infections. For example, while we prioritized antibiotics with standard dosing frequencies of ≤ twice daily, it remains possible that other frequencies would be optimal for dalbavancin synergy. Furthermore, the impact of cefadroxil and other well-tolerated orally bioavailable agents on the emergence of resistance to dalbavancin should also be evaluated. Nevertheless, given the lack of more clearly indicated alternatives and the favorable safety, cost, and oral dosing profiles, this combination could be considered in patients receiving prolonged courses of dalbavancin for invasive MRSA infections with suboptimal source control.

## CONCLUSIONS

Given the high risk of resistance emergence from chronic suppressive therapy of nonremovable devices such as LVADs, the convenience of dalbavancin dosing should be weighed carefully against the likelihood of resistance and cross-resistance during therapy. If dalbavancin is used in such cases, the dose and interval should be carefully considered, and the addition of oral cefadroxil may enhance activity.
